# RBF Neural Network Sliding Mode Control for Passification of Nonlinear Time-Varying Delay Systems with Application to Offshore Cranes

**DOI:** 10.3390/s22145253

**Published:** 2022-07-13

**Authors:** Baoping Jiang, Dongyu Liu, Hamid Reza Karimi, Bo Li

**Affiliations:** 1 School of Electronic and Information Engineering, Suzhou University of Science and Technology, Suzhou 215009, China; bpjiang@usts.edu.cn (B.J.); dyliuu@163.com (D.L.); 2 Department of Mechanical Engineering, Politecnico di Milano, 20156 Milan, Italy; 3 Department of Computing, The Hong Kong Polytechnic University, Hong Kong 999077, China; comp-bo.li@polyu.edu.hk

**Keywords:** sliding mode control, time-varying delay, nonlinear systems, neural networks

## Abstract

This paper is devoted to studying the passivity-based sliding mode control for nonlinear systems and its application to dock cranes through an adaptive neural network approach, where the system suffers from time-varying delay, external disturbance and unknown nonlinearity. First, relying on the generalized Lagrange formula, the mathematical model for the crane system is established. Second, by virtue of an integral-type sliding surface function and the equivalent control theory, a sliding mode dynamic system can be obtained with a satisfactory dynamic property. Third, based on the RBF neural network approach, an adaptive control law is designed to ensure the finite-time existence of sliding motion in the face of unknown nonlinearity. Fourth, feasible easy-checking linear matrix inequality conditions are developed to analyze passification performance of the resulting sliding motion. Finally, a simulation study is provided to confirm the validity of the proposed method.

## 1. Introduction

In industrial applications and the physical world, time delays are very common, such as their existence in chemical reactor systems [1], automotive powertrain systems [2], mechanical systems [3], population dynamics [4] and so on. The physical phenomena that produce time delays are caused by the transmission of information, energy, or different masses in long distance. The stability analysis of physical systems with time-delay is usually divided into two categories: the delay-independent criteria and the delay-dependent criteria; The latter condition includes information on the degree of system delay, which results in simpler and more conservative delay-independent stability criteria, particularly for small delays, than the delay-dependent stability criteria. Since last century, many efforts have been devoted to time-delay systems. For physical systems with constant time-delay, the stability issue for neural networks of neutral-type through a new Lyapunov functional was proposed in [5]; By proposing distributed dynamic controllers, the consensus of heterogeneous linear multi-agents with arbitrarily large constant communication delays was addressed in [6]. For physical systems with time-varying delay, the robust stability for time-varying structural and uncertain systems was studied by designing a novel delay-dependent stability criterion in [7]; In [8], the problems of stability analysis and stabilization for Takagi-Sugeno fuzzy systems in a discrete-time domain was investigated by employing a fuzzy Lyapunov-Krasovskii functional. For physical systems with distributed time-delay, the mode-dependent state feedback $H_{\infty }$ robust control design for uncertain distributed delay systems with Markov switching parameters was investigated in [9]; An improved distributed delay-dependent criterion for stability analysis and stabilization of uncertain fractional distributed delay systems was studied in [10]; See [11,12,13] and references therein for more details. Therefore, the research of time-delay systems have always been a hot topic in both theory and application.

The sliding mode control (SMC) [14] has demonstrated great advantages in finite-time fast response, robustness and stability for complex nonlinear systems since its appearance. Therefore, it has witnessed wide employment of SMC in industrial, for instance, the robot manipulators [15], the electric circuits [16] and the unmanned marine vehicles [17], etc. Theoretically, the investigation of SMC was also rich, such as the issues of reaching law design, reducing of chattering effect and high-order SMC design. For example, in order to further optimize the dynamic performance of permanent magnet synchronous motor speed regulation system, an improved sliding mode reaching law was proposed in [18]; Ref. [19] proposed an SMC algorithm for quad rotor helicopter based on least square method to solve the input overdetermined problem; Ref. [20] discussed to what extent a high-order SMC could serve as an alternative to the traditional SMC. In the filed time-delay systems, the SMC has also shown its superiorities, for example, the SMC approach was used to solve the issue of adaptive control problems for uncertain delayed switched systems with abrupt actuator failures in [21]; In [22], an observer-based adaptive SMC strategy was proposed to study the stabilization of nonlinear stochastic delayed Markov jump systems; The SMC design for mixed $H_{\infty }$ and passivity performance of linear time-varying delay systems with uncertainties was investigated in [23]; More details can be checked in [24,25,26]. However, the physical plant often suffers from unknown nonlinearities due to complex circumstances, and most recent SMC strategies for nonlinear systems are conducted relying on the assumption that the nonlinearities are norm-bounded, which is sometimes inaccurate and conservative.

On the other hand, it is known that the neural network is an efficient method for the approximations of nonlinear functions, which has attracted a lot of attention in the automation community, see the robot manipulators, heat exchangers [27,28], etc. Thus, the study of neural network combing with SMC for nonlinear time-delay systems is an interesting issue. In addition, passivity reflects the basic properties of many physical systems in the real world, and is usually used to define the energy dissipation and transformation of the system. Moreover, it plays a key role in the process of system analysis and design, particularly for some high-order systems. For example, many real systems need passivity to ensure effective noise attenuation. Therefore, passive-based control methods have been popular in the physical community.

To synthesize the above analysis, this paper studies the problems of neural network passivity-based SMC design for a class of nonlinear time-varying delay systems and its application in offshore cranes. By establishing a time-delay mathematical model for the crane system, we propose an integral-type switching surface and RBF-based adaptive SMC law, which not only ensures finite-time existence of the sliding motion, but also guarantees the obtained sliding mode dynamics has an exponential stability and a passivity performance. The main contributions are concluded as follows: (1) A comprehensive mathematical model is established for the crane system toward passification analysis, which includes time-varying delay, unknown nonlinearity and external disturbance; (2) For the obtained sliding motion, a set of strict LMI conditions are established that could check the system’s exponential stability, minimum passivity performance and maximum decay rate; (3) By proposing an RBF neural network, the system’s unknown nonlinearity can be easily compensated by the designed adaptive SMC law, with which the finite-time reachability condition is also ensured. The remaining contents are organized as follows: Preliminaries are given in Section 2; The main results are proposed in Section 3; A numerical example is provided in Section 4, and the paper is concluded in Section 5.

**Notations:**0 represents a zero matrix; I represents an identity matrix; The minimum and maximum eigenvalues of a matrix A are defined by $\lambda_{\min}\left(A\right)$ and $\lambda_{\max}\left(A\right)$, respectively; tr{A} means the trace of matrix A; The symmetric positive definite matrix is defined by A>0; * is a symmetric element of symmetric matrix; He{A} denotes $A+A^{T}$.

## 2. Model Establishing and Problem Statement

**Figure 1:**
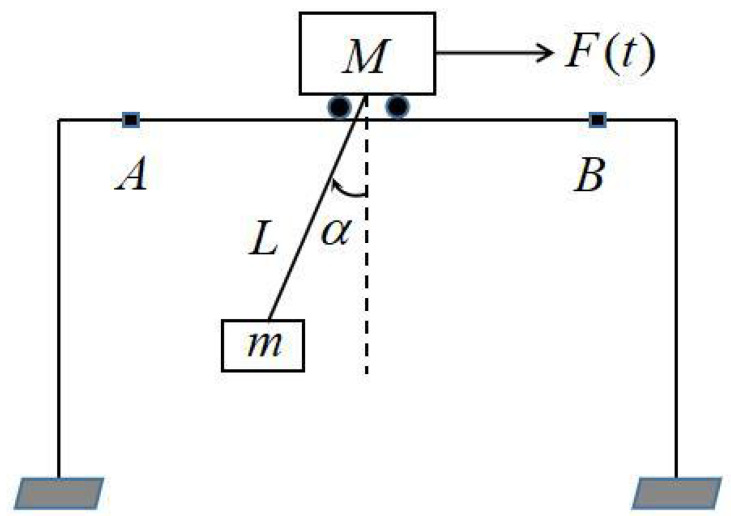
Abstract physical model of crane.

Crane, as a modern transfer equipment, has been widely used in modern factories, installation sites, container yards, indoor or outdoor warehouse loading and unloading, and transportation. The type offshore crane can be checked in [29], its equivalent physical model is simplified as shown in Figure 1, in which the load is attached to the cart by a cable; *m* is the load mass [kg], *M* is the cart mass [kg], *L* is the cable length [m], F(t) is the control input generated by the electric motor and θ is the load swing angle at the moment of movement. In real applications, the swing of loads is an important factor that disturbs the crane efficiency. In order to ensure that the load movement is smooth and steady, necessary efforts should be taken to deal with this issue. In general, the problem of fast transportation and positioning of heavy loads can be summarized as follows: Under the input F(t), the cart moves from position *A* to position *B* in the fastest time $t_{s}$ allowing the swing angle $|\theta (t_{s})|<\Delta$, in which Δ is the minimum allowable swing angle for the load in the process of movement.

**Figure 2:**
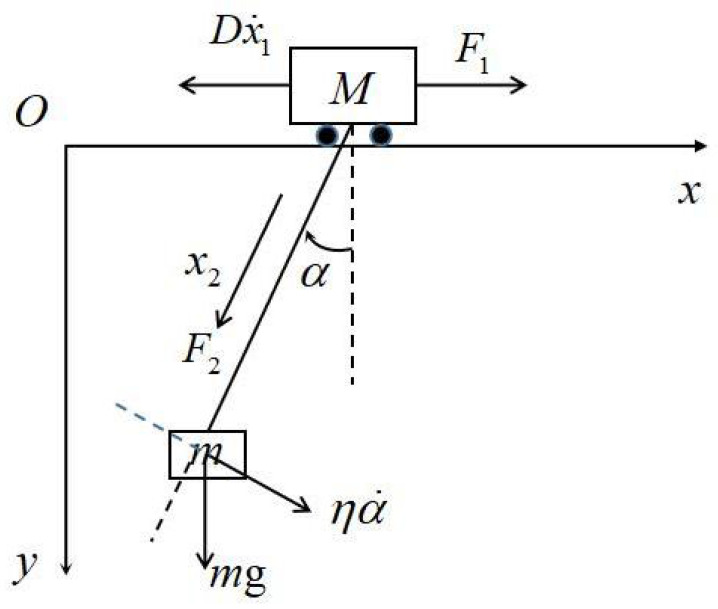
Physical model in a generalized coordinate.

To deal with this problem, let us establish the system mathematical model in a generalized coordinate; see Figure 2, in which $x_{1}$ is the position of the cart, $x_{2}$ is the cable length, α is the swing angle, $F_{1}$ is the pull imposed on the cart and $F_{2}$ is the lift for the load. *D* is the friction damping coefficient between the cart and the horizontal track, and η is the damping coefficient when the load swings. Therefore, the positions for the cart and the load in the generalized coordinate can be denoted by

(1) $$\left\{\begin{array}{c} x_{M}=x_{1}\\ y_{M}=0\\ x_{m}=x_{1}-x_{2}\sin\alpha \\ y_{m}=x_{2}\cos\alpha \end{array}\right.$$

Correspondingly, the velocity components for the cart and the load are obtained as

(2) $$\left\{\begin{array}{c} {\dot{x}}_{M}={\dot{x}}_{1}\\ {\dot{y}}_{M}=0\\ {\dot{x}}_{m}={\dot{x}}_{1}-x_{2}\dot{\alpha }\cos\alpha -{\dot{x}}_{2}\sin\alpha \\ {\dot{y}}_{m}={\dot{x}}_{2}\cos\alpha -x_{2}\dot{\alpha }\sin\alpha \end{array}\right.$$

On the other hand, it is known that the kinetic energy *T* of the overall system is denoted by

(3) $$\begin{array}{cc} \; & T=\frac{1}{2}MV_{M}^{2}+\frac{1}{2}mV_{m}^{2}\\ \; & \;=\frac{1}{2}M\left({\dot{x}}_{M}^{2}+{\dot{y}}_{M}^{2}\right)+\frac{1}{2}m\left({\dot{x}}_{m}^{2}+{\dot{y}}_{m}^{2}\right)\\ \; & \;=\frac{1}{2}\left(M+m\right){\dot{x}}_{1}^{2}+\frac{1}{2}\left({\dot{x}}_{2}^{2}+x_{2}^{2}{\dot{\alpha }}^{2}-2{\dot{x}}_{1}{\dot{x}}_{2}\sin\alpha -2{\dot{x}}_{1}x_{2}\dot{\alpha }\cos\alpha \right) \end{array}$$

Then, according to the generalized Lagrange formula that

(4) $$\frac{d}{dt}\left(\frac{\partial T}{\partial {\dot{q}}_{k}}\right)-\frac{\partial T}{\partial q_{k}}=F_{k},\;\left(k=1,2,...,n\right)$$

in which $q_{k}$ is the generalized coordinate, *n* is the degree of freedom and $F_{k}$ is the generalized force. Therefore, (4) yields

(5) $$\left\{\begin{array}{c} \frac{d}{dt}\left(\frac{\partial T}{\partial {\dot{x}}_{1}}\right)-\frac{\partial T}{\partial x_{1}}=F_{1}-D{\dot{x}}_{1}\\ \frac{d}{dt}\left(\frac{\partial T}{\partial {\dot{x}}_{2}}\right)-\frac{\partial T}{\partial x_{2}}=F_{2}+mg\cos\alpha \\ \frac{d}{dt}\left(\frac{\partial T}{\partial \dot{\alpha }}\right)-\frac{\partial T}{\partial \alpha }=-mgx_{2}\sin\alpha -\eta \dot{\alpha } \end{array}\right.$$

Further, the overall system model is obtained as follows:

(6) $$\left\{\begin{array}{c} \left(M+m\right){\ddot{x}}_{1}-m{\ddot{x}}_{2}\sin\alpha -2m{\dot{x}}_{2}\dot{\alpha }\cos\alpha -mx_{2}\ddot{\alpha }\cos\alpha +mx_{2}{\dot{\alpha }}^{2}\sin\alpha +D{\dot{x}}_{1}=F_{1}\\ m{\ddot{x}}_{2}-m{\ddot{x}}_{1}\sin\alpha -mg\cos\alpha -mx_{2}{\dot{\alpha }}^{2}=F_{2}\\ mx_{2}^{2}\ddot{\alpha }+2mx_{2}{\dot{x}}_{2}\dot{\alpha }-m{\ddot{x}}_{1}x_{2}\cos\alpha +mgx_{2}\sin\alpha +\eta \dot{\alpha }=0 \end{array}\right.$$

Regarding the dynamic model (6), linearization of the model in state-space is necessary for the purpose of stability analysis and control design. Considering that the length of cable is unchanged, that is $x_{2}=L=const.$, than ${\dot{x}}_{2}={\ddot{x}}_{2}=0$. Considering that the swing angle of the crane is quite small in the equilibrium point α=0 during the real movement process, so we can linearize the model with sinα≈α, cosα≈0 and ${\dot{\alpha }}^{2}\sin\alpha \approx 0$. In addition, it is deemed that the damping coefficient is quite small and taken as η=0, then the simplified model is taken as

(7) $$\left\{\begin{array}{c} \left(M+m\right){\ddot{x}}_{1}+D{\dot{x}}_{1}-mL\ddot{\alpha }=F_{1}\\ mL^{2}\ddot{\alpha }-mL{\ddot{x}}_{1}+mgL\alpha =0 \end{array}\right.$$

Now, define $z={\left[z_{1}\;z_{2}\;z_{3}\;z_{4}\right]}^{T}$, where $z_{1}=x_{1}$, $z_{2}={\dot{x}}_{1}$, $z_{3}=\alpha$ and $z_{4}=\dot{\alpha }$. Letting $x_{1}$ and α be the output variables, then the system (7) described in the state-space can be presented as:

(8) $$\left\{\begin{array}{c} \dot{z}=Az+Bu\\ y=Cz, \end{array}\right.$$

where u=F and

$$A=\left[\begin{array}{cccc} 0 & 1 & 0 & 0\\ 0 & -\frac{D}{M} & -\frac{mg}{M} & 0\\ 0 & 0 & 0 & 1\\ 0 & -\frac{D}{ML} & -\frac{(M+m)g}{ML} & 0 \end{array}\right],B=\left[\begin{array}{c} 0\\ \frac{1}{M}\\ 0\\ \frac{1}{ML} \end{array}\right],C=\left[\begin{array}{cccc} 1 & 0 & 0 & 0\\ 0 & 0 & 1 & 0 \end{array}\right],$$

Generally, let us consider a more complex environment in which the physical system suffers from unknown nonlinearity, external disturbance and time-varying state delays. Then, the description of state-space system will be denoted as

(9) $$\left\{\begin{array}{c} \dot{z}\left(t\right)=Az\left(t\right)+A_{\tau }z\left(t-\tau \left(t\right)\right)+B\left(u\left(t\right)+f\left(z\left(t\right)\right)\right)+B_{w}w\left(t\right)\\ y\left(t\right)=Cz\left(t\right)+D_{w}w\left(t\right),\\ z\left(s\right)=\varphi \left(s\right),\dot{z}\left(s\right)=\psi \left(s\right);\;s\in \left[-\tau_{1},0\right], \end{array}\right.$$

in which $z\left(t\right)\in R^{n}$ is the state variable, $u\left(t\right)\in R^{m}$ is the control variable and $y\left(t\right)\in R^{q}$ is the controlled output. The system matrices A, $A_{\tau }$, B, C, $B_{w}$ and $D_{w}$ are with appropriate dimensions, and B is full column rank. The system unknown nonlinearity is f(z(t)) and $w\left(t\right)\in L_{2}\left[0,+\infty \right)$ denotes the norm-bounded external disturbance. The system state delay is represented as τ(t), satisfying

(10) $$0\leq \tau \left(t\right)\leq \tau_{1},\;\dot{\tau }\left(t\right)\leq \tau_{2}<1,$$

in which $\tau_{1}$ and $\tau_{2}$ are known constants.

The following definition and lemma are useful in the following analysis.

**Definition** **1.** ([30,31]). *The nonlinear system (9) is said to have a passification performance index γ, if the following two conditions are satisfied:*

*(1) With w(t)=0, the solution z(t) of the system (9) is internally exponentially stable, i.e,*

(11) $$∥z\left(t\right)∥\leq \delta e^{-\beta t}{∥\phi )∥}_{\tau },$$

*in which δ≥1, β>0 is the decay rate and ${∥\phi ∥}_{\tau }={sup}_{s\in [-\tau ,0]}\sqrt{\varphi^{2}\left(s\right)+\psi^{2}\left(s\right)}$.*

*(2) For nonzero $w\left(t\right)\in L_{2}\left[0,+\infty \right)$, the following inequality is satisfied under zero initial condition,*

(12) $$2\int_{0}^{t}w^{T}\left(s\right)y\left(s\right)ds\geq -\gamma \int_{0}^{t}w^{T}\left(s\right)w\left(s\right)ds,$$

*in which γ is a positive constant and for all t≥0.*

**Lemma** **1.** ([32]). *For a given matrix satisfying $0<P\in R^{n\times n}$, and a differentiable vector function ζ(t) has appropriate dimensions. Then, it holds*

(13) $${\left[\int_{t-\tau (t)}^{t}\dot{\zeta }\left(s\right)ds\right]}^{T}P\left[\int_{t-\tau (t)}^{t}\dot{\zeta }\left(s\right)ds\right]\leq \tau_{1}\int_{t-\tau (t)}^{t}{\dot{\zeta }}^{T}\left(s\right)P\dot{\zeta }\left(s\right)ds.$$

## 3. Main Results

This section will propose an SMC strategy so as to ensure the closed-loop system has a passification performance with an exponential stability property. The steps here include: design of sliding surface, passification analysis and RBF neural network sliding mode controller design.

### 3.1. Sliding Motion Design

For the system (9), let us define the following integral-type switching hyperplane function

(14) $$s\left(z,t\right)=Gz\left(t\right)-\int_{0}^{t}G\left(A+BK\right)z\left(s\right)ds-\int_{0}^{t}GA_{\tau }z\left(s-\tau \left(s\right)\right)ds,$$

where G and K are both real matrices to be designed. Particularly, it is required that GB is nonsingular.

According to the dynamics of the system (9), the solution z(t) follows

(15) $$z\left(t\right)=z\left(0\right)+\int_{0}^{t}\left[Az\left(s\right)+A_{\tau }z\left(s-\tau \left(s\right)\right)+B\left(u\left(s\right)+f\left(z\left(t\right)\right)\right)+B_{w}w\left(s\right)\right]ds.$$

Combining (14) with (15), it is obtained that

(16) $$\begin{array}{cc} \; & s\left(z,t\right)=G\left[z\left(0\right)+\int_{0}^{t}\left[Az\left(s\right)+A_{\tau }z\left(s-\tau \left(s\right)\right)+B\left(u\left(s\right)+f\left(z\left(t\right)\right)\right)+B_{w}w\left(s\right)\right]ds\right]\\ \; & \;-\int_{0}^{t}G\left(A+BK\right)z\left(s\right)ds-\int_{0}^{t}GA_{\tau }z\left(s-\tau \left(s\right)\right)ds. \end{array}$$

According to the SMC theory [14], it holds that both s(z,t)=0 and $\dot{s}\left(z,t\right)=0$ when the sliding surface s(z,t)=0 is reached. Therefore, using the condition $\dot{s}\left(z,t\right)=0$, one can derive an equivalent control variable:

(17) $$u_{eq}\left(t\right)=Kz\left(t\right)-{\left(GB\right)}^{-1}BB_{w}w\left(t\right)-f\left(z\left(t\right)\right).$$

By substituting (7) into the system (9), one can obtain the following sliding mode dynamic (SMD) system

(18) $$\dot{z}\left(t\right)=\left(A+BK\right)z\left(t\right)+A_{\tau }z\left(t-\tau \left(t\right)\right)+{\tilde{B}}_{w}w\left(t\right),$$

in which ${\tilde{B}}_{w}=\left[I-B{\left(GB\right)}^{-1}B^{T}\right]B_{w}$.

**Remark** **1.** *An integral sliding surface function of the form (14) is proposed, which contains an integral term with time delay. The advantage of such a sliding surface is that, since the design of the memory controller is more complicated in practice, it will be more convenient in the subsequent controller design.*

**Figure 3:**
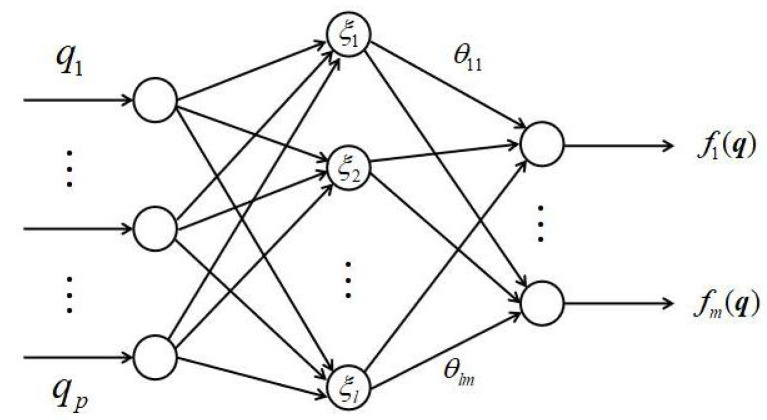
The RBF network structure.

In the sequel, an SMC law will be presented to ensure the finite-time existence of sliding motion on the sliding surface s(z,t)=0. However, due to f(z(t)) being an unknown function, how to compensate the effect of f(z(t)) in the whole phase should be considered first. In the following, a radial basis function $\theta^{T}\xi \left(z\right)$ is applied to estimate the unknown function f(·), in which $\theta \in R^{l\times m}$, $\xi \left(q\right)\in R^{l}$ is a vector-valued function and $q\in R^{p}$ is the input vector of the RBF neural network. The structure of three-layer RBF network is presented in Figure 3.

**Lemma** **2.** *For a compact set $\Omega_{q}\in R^{p}$, $q\in \Omega_{q}\in R^{p}$, $\xi \left(q\right)={\left[\xi_{1}\left(q\right)\;\xi_{2}\left(q\right)\;\cdots \xi_{l}\left(q\right)\right]}^{T}$ is the Gaussian basis function vector with the form*

$$\xi_{j}\left(q\right)=exp\left[-\frac{∥q-m_{j}{∥}^{2}}{2\sigma_{j}^{2}}\right],\sigma_{j}\geq 0,j=1,2,\ldots ,l.$$

*where $m_{j}\in R^{p}$ and $\sigma_{j}\in R$ represent the center and width of above Gaussian function, respectively. If the integer l is chosen to be as large as possible, then there exists a $\theta^{*}\in R^{l\times m}$ such that*

$$f\left(q\right)=\theta^{*T}\xi \left(q\right)+\delta^{*}\left(q\right),$$

*in which $∥\delta^{*}\left(q\right)∥\leq \delta$, and δ is a known constant.*

In the following, $\hat{\theta }\left(t\right)$ is used to denote the estimation of $\theta^{*}$. The corresponding error is denoted by $\tilde{\theta }\left(t\right)=\hat{\theta }\left(t\right)-\theta^{*}$.

**Theorem** **1.** *Given the nonlinear system (9), we define its switching surface function in (14). Then, the SMC law designed below could ensure the state trajectories driven onto the proposed sliding surface s(z,t)=0 in finite time,*

(19) $$\begin{array}{cc} \; & u\left(t\right)=Kz\left(t\right)-{\hat{\theta }}^{T}\left(t\right)\xi \left(q\right)-(∥{\left(B^{T}B\right)}^{-1}B{\tilde{B}}_{w}∥∥w\left(t\right)∥+\rho )sgn\left(s\left(z,t\right)\right)], \end{array}$$

*in which sgn(·) is the symbolic function, ρ is chosen such that ρ−δ≥ε>0, and $\dot{\hat{\theta }}\left(t\right)=\Lambda \xi \left(q\right)s^{T}\left(z,t\right)$ with *Λ* is a known matrix parameter.*

**Proof.**  Choose $G=B^{T}$ and the Lyapunov function candidate below:

(20) $$V\left(t\right)=\frac{1}{2}\left[s^{T}\left(z,t\right){\left(B^{T}B\right)}^{-1}s\left(z,t\right)+tr\left\{{\tilde{\theta }}^{T}\left(t\right)\Lambda^{-1}\tilde{\theta }\left(t\right)\right\}\right].$$

Then, it obtains for (16) that

(21) $$\begin{array}{cc} \; & \dot{V}\left(t\right)=s^{T}\left(z,t\right){\left(B^{T}B\right)}^{-1}\dot{s}\left(z,t\right)+tr\left\{{\tilde{\theta }}^{T}\left(t\right)\Lambda^{-1}\dot{\tilde{\theta }}\left(t\right)\right\}\\ \; & \;=s^{T}\left(z,t\right)\left[Kz\left(t\right)+\left(u\left(t\right)+f\left(z\left(t\right)\right)\right)\right]+s^{T}\left(z,t\right){\left(B^{T}B\right)}^{-1}B{\tilde{B}}_{w}w\left(t\right)\\ \; & \;-s^{T}\left(z,t\right){\hat{\theta }}^{T}\left(t\right)\xi \left(q\right)+tr\left\{{\tilde{\theta }}^{T}\left(t\right)\Lambda^{-1}\dot{\tilde{\theta }}\left(t\right)\right\}\\ \; & \;\leq -s^{T}\left(z,t\right)[(∥{\left(B^{T}B\right)}^{-1}B{\tilde{B}}_{w}∥∥w\left(t\right)∥+\rho )sgn\left(s\left(z,t\right)\right)-f\left(z\left(t\right)\right)]-s^{T}\left(z,t\right){\hat{\theta }}^{T}\left(t\right)\xi \left(q\right)\\ \; & \;+∥s\left(z,t\right)∥{\left(B^{T}B\right)}^{-1}B{\tilde{B}}_{w}∥∥w\left(t\right)∥+tr\left\{\left({\hat{\theta }}^{T}\left(t\right)-\theta^{*T}\right)\Lambda^{-1}\dot{\tilde{\theta }}\left(t\right)\right\} \end{array}$$

In view of $\dot{\tilde{\theta }}\left(t\right)=\dot{\hat{\theta }}\left(t\right)$ and the property that tr{AB}=tr{BA}, it is seen in (21) that

(22) $$\begin{array}{cc} \; & \Gamma =-s^{T}\left(z,t\right)\left[\rho sgn\left(s\left(z,t\right)\right)-f\left(z\left(t\right)\right)\right]-s^{T}\left(z,t\right){\hat{\theta }}^{T}\left(t\right)\xi \left(q\right)\\ \; & \;+tr\{\left({\hat{\theta }}^{T}\left(t\right)-\theta^{*T}\right)\Lambda^{-1}\dot{\tilde{\theta }}\left(t\right)\}\\ \; & \;\leq -s^{T}\left(z,t\right)\left[\rho sgn\left(s\left(z,t\right)\right)-\delta^{*}\left(z\right)-\theta^{*T}\xi \left(q\right)\right]-s^{T}\left(z,t\right){\hat{\theta }}^{T}\left(t\right)\xi \left(z\right)\\ \; & \;+tr\{\left({\hat{\theta }}^{T}\left(t\right)-\theta^{*T}\right)\xi \left(q\right)s^{T}\left(z,t\right)\}\\ \; & \;\leq -tr\left\{s^{T}\left(z,t\right)\left(\hat{\theta }\left(t\right)-\theta^{*}\right)\xi \left(q\right)\right\}+tr\left\{\left({\hat{\theta }}^{T}\left(t\right)-\theta^{*T}\right)\xi \left(q\right)s^{T}\left(z,t\right)\right\}\\ \; & \;-\left(\rho -\delta \right)s^{T}\left(z,t\right)sgn\left(s\left(z,t\right)\right)\\ \; & \;\leq -\varepsilon s^{T}\left(z,t\right)sgn\left(s\left(z,t\right)\right) \end{array}$$

Therefore, by substituting (19) into (21) and in view of ∥s(z,t)∥≤|s(z,t)|. One can read from (21) and (22) that

(23) $$\dot{V}\left(t\right)\leq -\varepsilon ∥s\left(z,t\right)∥\leq 0.$$

Thus, the finite-time reachability condition is satisfied. This covers the proof. □

### 3.2. Stability Analysis

**Theorem** **2.** *For given positive scalars α>0 and γ>0, if it finds matrices X>0, $Q_{1}>0$, $Q_{2}>0$ and proper matrix $S_{i}$ (i=1,2,3,4) that meets the condition below*

(24) $$\Theta =\left[\begin{array}{cccc} \Theta_{11} & \Theta_{12} & S_{1}A_{\tau }-S_{3}^{T} & S_{1}{\tilde{B}}_{w}\\ {}* & \Theta_{22} & \Theta_{23} & S_{2}{\tilde{B}}_{w}-C^{T}\\ {}* & * & \Theta_{33} & S_{3}{\tilde{B}}_{w}\\ {}* & * & * & -\gamma I-D_{w}-D_{w}^{T} \end{array}\right]<0,$$

*where*

$\Theta_{11}=-S_{1}-S_{1}^{T}+\tau_{1}^{2}Q_{2},$

$\Theta_{12}=S_{1}\left(A+BK\right)-S_{2}^{T}+X,$

$\Theta_{22}=He\left\{S_{2}\left(A+BK\right)\right\}+\alpha X+Q_{1}-e^{-\alpha \tau_{1}}Q_{2},$

$\Theta_{23}=S_{2}A_{\tau }+{\left(A+BK\right)}^{T}S_{3}^{T}+e^{-\alpha \tau_{1}}Q_{2},$

$\Theta_{33}=-\left(1-\tau_{2}\right)e^{-\alpha \tau_{1}}Q_{1}-e^{-\alpha \tau_{1}}Q_{2}+He\left\{S_{3}A_{\tau }\right\},$

*Then, the SMD system (18) satisfies the properties in Definition 1 with exponential stability and passification performance index γ.*

**Proof.**  First, let us check the exponential stability of the SMD system (18) for the case w(t)=0. Now, consider the Lyapunov functional candidate below:

(25) $$V\left(t\right)=V_{1}\left(t\right)+V_{2}\left(t\right)+V_{3}\left(t\right),$$

in which

$V_{1}\left(t\right)=z^{T}\left(t\right)Xz\left(t\right)$,

$V_{2}\left(t\right)=\int_{t-\tau (t)}^{t}e^{-\alpha (t-s)}z^{T}\left(s\right)Q_{1}z\left(s\right)ds,$

$V_{3}\left(t\right)=\tau_{1}\int_{-\tau_{1}}^{0}\int_{t+\theta }^{t}e^{-\alpha (t-s)}{\dot{z}}^{T}\left(s\right)Q_{2}\dot{z}\left(s\right)dsd\theta .$

Then, calculating the derivative of V(t) along the trajectories of the SMD system (18) yields

(26) $${\dot{V}}_{1}\left(t\right)=2z^{T}\left(t\right)X\dot{z}\left(t\right),$$

(27) $$\begin{array}{cc} \; & {\dot{V}}_{2}\left(t\right)=z^{T}\left(t\right)Q_{1}z\left(t\right)-\left(1-\dot{\tau }\left(t\right)\right)e^{-\alpha \tau (t)}z^{T}\left(t-\tau \left(t\right)\right)Q_{1}z\left(t-\tau \left(t\right)\right)-\alpha V_{2}\left(t\right)\\ \; & \;\leq z^{T}\left(t\right)Q_{1}z\left(t\right)-\left(1-\tau_{2}\right)e^{-\alpha \tau_{1}}z^{T}\left(t-\tau \left(t\right)\right)Q_{1}z\left(t-\tau \left(t\right)\right)-\alpha V_{2}\left(t\right), \end{array}$$

(28) $$\begin{array}{cc} \; & {\dot{V}}_{3}\left(t\right)\leq \tau_{1}^{2}{\dot{z}}^{T}\left(s\right)Q_{2}\dot{z}\left(s\right)-\tau_{1}\int_{t-\tau_{1}}^{t}e^{-\alpha (t-s)}{\dot{z}}^{T}\left(s\right)Q_{2}\dot{z}\left(s\right)ds-\alpha V_{3}\left(t\right). \end{array}$$

in which it is seen that

$$\begin{array}{cc} \; & -\tau_{1}\int_{t-\tau_{1}}^{t}e^{-\alpha (t-s)}{\dot{z}}^{T}\left(s\right)Q_{2}\dot{z}\left(s\right)ds\leq -\tau_{1}e^{-\alpha \tau_{1}}\int_{t-\tau (t)}^{t}e^{-\alpha (t-s)}{\dot{z}}^{T}\left(s\right)Q_{2}\dot{z}\left(s\right)ds\\ \; & \;\leq -e^{-\alpha \tau_{1}}{\left[\int_{t-\tau (t)}^{t}\dot{z}\left(s\right)ds\right]}^{T}Q_{2}\left[\int_{t-\tau (t)}^{t}\dot{z}\left(s\right)ds\right]\\ \; & \;=-e^{-\alpha \tau_{1}}{\left[z\left(t\right)-z\left(t-\tau \left(t\right)\right)\right]}^{T}Q_{2}\left[z\left(t\right)-z\left(t-\tau \left(t\right)\right)\right]. \end{array}$$

Particularly, it is easily obtained from the SMD system (18) that the following equation holds

(29) $$2\eta^{T}\left(t\right)S\left[\left(A+BK\right)z\left(t\right)+A_{\tau }z\left(t-\tau \left(t\right)\right)+{\tilde{B}}_{w}w\left(t\right)-\dot{z}\left(t\right)\right]=0,$$

in which $\eta \left(t\right)={\left[{\dot{z}}^{T}\left(t\right)\;\;z^{T}\left(t\right)\;\;z^{T}\left(t-\tau \left(t\right)\right)\right]}^{T}$, and $S={\left[S_{1}^{T}\;S_{2}^{T}\;S_{3}^{T}\right]}^{T}$.

Combing (26)–(29), it obtains

(30) $$\dot{V}\left(t\right)\leq \eta^{T}\left(t\right)\bar{\Theta }\eta \left(t\right)-\alpha V\left(t\right),$$

in which

$$\bar{\Theta }=\left[\begin{array}{ccc} \Theta_{11} & \Theta_{12} & S_{1}A_{\tau }-S_{3}^{T}\\ {}* & {\tilde{\Theta }}_{22} & \Theta_{23}\\ {}* & * & \Theta_{33} \end{array}\right],$$

with ${\tilde{\Theta }}_{22}=He\left\{S_{2}\left(A+BK\right)\right\}+\alpha X+Q_{1}-e^{-\alpha \tau_{1}}Q_{2}.$

Applying (24), one can read $\dot{V}\left(t\right)+\alpha V\left(t\right)\leq \eta^{T}\left(t\right)\bar{\Theta }\eta \left(t\right)<0$ for η(t)≠0. Integrating both sides of this inequality from 0 to *t*. Then one derives that

(31) $$V\left(t\right)<e^{-\alpha t}V\left(0\right).$$

Recalling (25), we know there is a constant $\lambda_{1}$ that meets $0<\lambda_{1}\leq \lambda_{\min}\left(X\right)$ so as to

(32) $$\lambda_{1}{∥z\left(t\right)∥}^{2}<V\left(t\right).$$

Further, denoting $\lambda_{2}=\lambda_{\max}\left(X\right)$, $\lambda_{3}=\lambda_{\max}\left(Q_{1}\right)$ and $\lambda_{4}=\lambda_{\max}\left(Q_{2}\right)$. Then, it is easily seen that

(33) $$V\left(0\right)\leq \lambda_{2}{∥z\left(0\right)∥}^{2}+\lambda_{3}\int_{-\tau (0)}^{0}e^{\alpha s}∥z^{T}{\left(s\right)∥}^{2}ds+\tau_{1}\lambda_{4}\int_{-\tau_{1}}^{0}\int_{\theta }^{0}e^{\alpha s}{∥{\dot{z}}^{T}\left(s\right)∥}^{2}dsd\theta .$$

Thus, one can obtain the following result from (31)–(33) that

(34) $$\lambda_{1}{∥z\left(t\right)∥}^{2}\leq V_{1}\left(t\right)\leq V\left(t\right)\leq e^{-\alpha t}V\left(0\right)\leq \lambda e^{-\alpha t}{∥\phi ∥}_{\tau }^{2}$$

in which $\lambda =\lambda_{2}+\frac{\lambda_{3}}{\alpha }\left(1-e^{-\alpha \tau_{1}}\right)+\frac{\tau_{1}\lambda_{4}}{\alpha }\left[\tau_{1}-\frac{1}{\alpha }\left(1-e^{-\alpha \tau_{1}}\right)\right]$. At this moment, it is seen that the SMD system (18) is exponentially stable.

Next, let us check the passivity performance, denoted by

(35) $$J=\int_{0}^{t}\left[-2w^{T}\left(s\right)y\left(s\right)-\gamma w^{T}\left(s\right)w\left(s\right)\right]ds.$$

Under zero-initial condition, it is seen that $\int_{0}^{t}\dot{V}\left(s\right)ds=V\left(t\right)-V\left(0\right)>0$, then

(36) $$\begin{array}{cc} \; & J\leq \int_{0}^{t}\left[-2w^{T}\left(s\right)y\left(s\right)-\gamma w^{T}\left(s\right)w\left(s\right)+\dot{V}\left(s\right)+\alpha V\left(s\right)\right]ds\\ \; & \;=\int_{0}^{t}{\bar{\eta }}^{T}\left(s\right)\Theta \bar{\eta }\left(s\right)ds, \end{array}$$

in which $\bar{\eta }\left(t\right)={\left[{\dot{z}}^{T}\left(t\right)\;\;z^{T}\left(t\right)\;\;z^{T}\left(t-\tau \left(t\right)\right)\;\;w^{T}\left(t\right)\right]}^{T}$.

Since Θ<0, it derives from (36) that

(37) $$\dot{V}\left(t\right)+\alpha V\left(t\right)\leq 2w^{T}\left(t\right)y\left(t\right)+\gamma w^{T}\left(t\right)w\left(t\right).$$

Now, integrating (37) from both sides in the region 0 to *t* results in

(38) $$\int_{0}^{t}e^{\alpha s}V\left(s\right)ds\leq \int_{0}^{t}e^{\alpha s}\left[2w^{T}\left(s\right)y\left(s\right)+\gamma w^{T}\left(s\right)w\left(s\right)\right]ds,$$

which means

(39) $$\begin{array}{cc} \; & V\left(t\right)\leq \int_{0}^{t}e^{-\alpha (t-s)}\left[2w^{T}\left(s\right)y\left(s\right)+\gamma w^{T}\left(s\right)w\left(s\right)\right]ds,\\ \; & \;\leq \int_{0}^{t}\left[2w^{T}\left(s\right)y\left(s\right)+\gamma w^{T}\left(s\right)w\left(s\right)\right]ds. \end{array}$$

In (39), letting t→+∞, then it obtains $2\int_{0}^{t}w^{T}\left(s\right)y\left(s\right)ds\geq -\gamma \int_{0}^{t}w^{T}\left(s\right)w\left(s\right)ds$. Hence, the passification performance is guaranteed. This completes the proof. □

### 3.3. Computation of Gain Matrix

In Theorem 2, an exponential stability criterion for the SMD system (18) with a decay rate α is established. However, our attention is to design a feasible control gain matrix K for exponential stability and passification performance of closed-loop system. Therefore, a theorem is further proposed as follows.

**Theorem** **3.** *Given positive scalars $\epsilon_{i}\left(i=1,2,3\right)$, α>0 and γ>0, if it finds matrices $\bar{X}>0$, ${\bar{Q}}_{1}>0$, ${\bar{Q}}_{2}>0$ and proper matrices Y and Y such that the following condition holds*

(40) $$\Phi =\left[\begin{array}{cccc} \Phi_{11} & \Phi_{12} & \epsilon_{1}A_{\tau }Y^{T}-\epsilon_{3}Y & \epsilon_{1}{\tilde{B}}_{w}\\ {}* & \Phi_{22} & \Phi_{23} & \epsilon_{2}{\tilde{B}}_{w}-YC^{T}\\ {}* & * & \Phi_{33} & \epsilon_{3}{\tilde{B}}_{w}\\ {}* & * & * & -\gamma I-D_{w}-D_{w}^{T} \end{array}\right]<0,$$

*where*

$\Phi_{11}=-\epsilon_{1}\left(Y+Y^{T}\right)+\tau_{1}^{2}{\bar{Q}}_{2},$

$\Phi_{12}=\epsilon_{1}\left(AY^{T}+BY\right)-\epsilon_{2}Y^{T}+\bar{X},$

$\Phi_{22}=He\left\{\epsilon_{2}\left(AY^{T}+BY\right)\right\}+\alpha \bar{X}+{\bar{Q}}_{1}-e^{-\alpha \tau_{1}}{\bar{Q}}_{2},$

$\Phi_{23}=\epsilon_{2}A_{\tau }Y^{T}+\epsilon_{3}{\left(AY^{T}+BY\right)}^{T}+e^{-\alpha \tau_{1}}{\bar{Q}}_{2},$

$\Phi_{33}=-\left(1-\tau_{2}\right)e^{-\alpha \tau_{1}}{\bar{Q}}_{1}-e^{-\alpha \tau_{1}}{\bar{Q}}_{2}+He\left\{\epsilon_{3}A_{\tau }Y^{T}\right\}.$

*Then, the SMD system (18) satisfies the properties in Definition 1 with exponential stability and passification performance γ. Besides, the controller gain matrix is computed as $K=YY^{-T}$.*

**Proof.**  Letting $S_{i}=\epsilon_{i}Y^{-1}\left(i=1,2,3\right)$ with each $\epsilon_{i}$ being given. Then, substituting them into (24) and conducting a congruence transformation to (24) by diag{Y,Y,Y,I}. At the same time, define $\bar{X}=YXY^{T}$, ${\bar{Q}}_{1}=YQ_{1}Y^{T}$ and ${\bar{Q}}_{2}=YQ_{2}Y^{T}$, we have Φ<0 in (40), where the matrix Y is set by $Y=KY^{T}$. □

**Remark** **2.** *In Theorems 2 and 3, both delay-dependent and delay-derivative-dependent exponential stability criteria for the SMD system (18) are established, which bring the following advantages: (1) It is seen that these conditions are not only fit for constant time-delay systems, but also available for the time-varying case; (2) The LMI conditions are easily changed into the asymptotic stability criteria with the decay rate α=0; (3) The condition is supposed to be less conservative compared with some recent results since a set of free weighting matrices are introduced.*

As we know, to have a fast convergence of system state trajectories requires a relatively large value of decay rate. Thus, it is desired to allow a maximal α to ensure the exponential stability based on the above Theorem 3. To this end, we propose a Corollary below.

**Corollary** **1.** *For given positive scalars α>0, γ>0 and $\epsilon_{i}\left(i=1,2,3\right)$, if it finds matrices $\bar{X}>0$, ${\bar{Q}}_{i}>0$ (i=1,2), $K_{j}>0$ (j=1,2,3), and proper matrices Y and Y satisfy the following conditions*

minβ

s.t.

(41) $$\Xi =\left[\begin{array}{cccc} \Xi_{11} & \Xi_{12} & \epsilon_{1}A_{\tau }Y^{T}-\epsilon_{3}Y & \epsilon_{1}{\tilde{B}}_{w}\\ {}* & \Xi_{22} & \Xi_{23} & \epsilon_{2}{\tilde{B}}_{w}-YC^{T}\\ {}* & * & \Xi_{33} & \epsilon_{3}{\tilde{B}}_{w}\\ {}* & * & * & -\gamma I-D_{w}-D_{w}^{T} \end{array}\right]<0,$$

(42) $$\bar{X}<\beta K_{1},$$

(43) $$\tau_{1}K_{2}<\beta \left({\bar{Q}}_{1}-K_{2}\right),$$

(44) $$\tau_{1}K_{3}<\beta \left({\bar{Q}}_{2}-K_{3}\right),$$

*where*

$\Xi_{11}=\epsilon_{1}\left(Y+Y^{T}\right)+\tau_{1}^{2}{\bar{Q}}_{2},$

$\Xi_{12}=\epsilon_{1}\left(AY^{T}+BH\right)-\epsilon_{2}Y^{T}+\bar{X},$

$\Xi_{22}=He\left\{\epsilon_{2}\left(AY^{T}+BH\right)\right\}+K_{1}+{\bar{Q}}_{1}-K_{3},$

$\Xi_{23}=\epsilon_{2}A_{\tau }+\epsilon_{3}{\left(AY^{T}+\bar{B}Y\right)}^{T}+K_{3},$

$\Xi_{33}=-\left(1-\tau_{2}\right)K_{2}-K_{3}+He\left\{\epsilon_{3}A_{\tau }Y^{T}\right\}.$

*Then, the SMD system (18) satisfies the properties in Definition 1 with exponential stability and passification performance γ.*

**Proof.**  Define $K_{1}>\alpha \bar{X}$, $K_{2}=e^{-\alpha \tau_{1}}{\bar{Q}}_{1}$ and $K_{3}=e^{-\alpha \tau_{1}}{\bar{Q}}_{2}$. By setting $\beta =\alpha^{-1}$, one can obtain

$$\bar{X}<\beta K_{1};$$

$$K_{2}=e^{-\alpha \tau_{1}}{\bar{Q}}_{1}\Rightarrow \left(1+\alpha \tau_{1}\right)K_{2}<e^{\alpha \tau_{1}}K_{2}={\bar{Q}}_{1}\Rightarrow \tau_{1}K_{2}<\beta \left({\bar{Q}}_{1}-K_{2}\right);$$

$$K_{3}=e^{-\alpha \tau_{1}}{\bar{Q}}_{2}\Rightarrow \left(1+\alpha \tau_{1}\right)K_{3}<e^{\alpha \tau_{1}}K_{3}={\bar{Q}}_{2}\Rightarrow \tau_{1}K_{3}<\beta \left({\bar{Q}}_{2}-K_{3}\right);$$

This covers the proof. □

**Figure 4:**
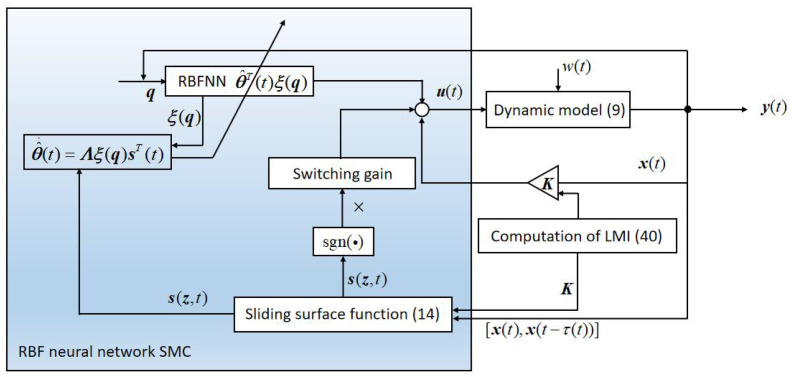
Structure of the controller.

Overall, the above Figure 4 shows how the proposed controller is implemented with necessary steps for the considered system. It should be noted that the controller gain matrix K in Figure 4 is computed by LMI (40) off-line in advance, then we initialize corresponding parameters to implement the controller.

## 4. Simulation Study

In this part, an example with a simulation study is provided for the crane system. Letting M=200, m=100, L=4, D=0.1 and g=9.81. Therefore, we can consider the following system parameters for the system (9):

$$A=\left[\begin{array}{cccc} 0 & 1 & 0 & 0\\ 0 & -0.0005 & -4.905 & 0\\ 0 & 0 & 0 & 1\\ 0 & -0.000125 & -3.67875 & 0 \end{array}\right],A_{\tau }=\left[\begin{array}{cccc} -0.1 & 0 & 0.2 & 0\\ 0 & 0.1 & 0 & -0.1\\ 0 & 0.2 & 0 & 0\\ -0.2 & 0 & 0.3 & 0 \end{array}\right],$$

$$B=\left[\begin{array}{c} 0\\ 0.005\\ 0\\ 0.00125 \end{array}\right],B_{w}=\left[\begin{array}{cc} 1.0 & 0\\ 0 & 0.1\\ 0.5 & 0\\ 0 & 1 \end{array}\right],C=\left[\begin{array}{cccc} 1 & 0 & 0 & 0\\ 0 & 0 & 1 & 0 \end{array}\right],D_{w}=\left[\begin{array}{cc} 0.1 & -0.1\\ 0.1 & -0.1 \end{array}\right],$$

Assuming that the system subjects to the nonlinearity by $f\left(z\left(t\right)\right)=0.1sin\left(z_{1}\left(t\right)\right)$. The system state-delay is chosen as τ(t)=0.2 such that $\tau_{1}=0.2$ and $\tau_{2}=0$ can be easily obtained. Our aims here are that: (1) To compute the controller gain matrix K so as to ensure the SMD system (18) is exponentially stable and satisfies a passivity performance index γ; (2) To design an adaptive SMC law given in (19) so as to ensure finite-time existence of desired sliding motion. For computation, define $G=B^{T}$, select scalars $\epsilon_{i}=1\left(i=1,2,3\right)$, the decay rate α=0.1 and γ=2.5. According to Theorem 3, and by solving the condition (40), one can obtain feasible solutions as follows:

$$\bar{X}=\left[\begin{array}{cccc} 9.5049 & -0.9422 & -2.7456 & 6.0718\\ -0.9422 & 29.5851 & 0.6763 & 7.3036\\ -2.7456 & 0.6763 & 4.0419 & -2.3185\\ 6.0718 & 7.3036 & -2.3185 & 17.4198 \end{array}\right],$$

$${\bar{Q}}_{1}=\left[\begin{array}{cccc} 4.4312 & -3.3115 & -2.5993 & 1.6767\\ -3.3115 & 33.2919 & 10.4344 & 12.1907\\ -2.5993 & 10.4344 & 8.3995 & 1.9303\\ 1.6767 & 12.1907 & 1.9303 & 12.5318 \end{array}\right],$$

$${\bar{Q}}_{2}=\left[\begin{array}{cccc} 17.6999 & -0.6692 & 4.1431 & 4.3860\\ -0.6692 & 29.5281 & 1.0937 & 1.9281\\ 4.1431 & 1.0937 & 22.8592 & 3.2521\\ 4.3860 & 1.9281 & 3.2521 & 14.5217 \end{array}\right],$$

$$Y=\left[\begin{array}{cccc} 3.3517 & -2.5691 & -0.4337 & 3.6388\\ -0.3088 & 5.2287 & -1.8335 & -1.5211\\ 0.8571 & 3.5562 & 1.2885 & -1.8051\\ 0.4697 & -1.5791 & 2.4504 & 3.4195 \end{array}\right],$$

$$Y=10^{3}\times \left[-1.1127\;\;-7.6417\;\;2.5487\;\;-0.0582\right].$$

Based on the above solutions, the controller gain matrix can be computed as:

$$K=10^{3}\times \left[-0.4401\;\;-1.7664\;\;-0.0237\;\;-0.3470\right].$$

**Figure 5:**
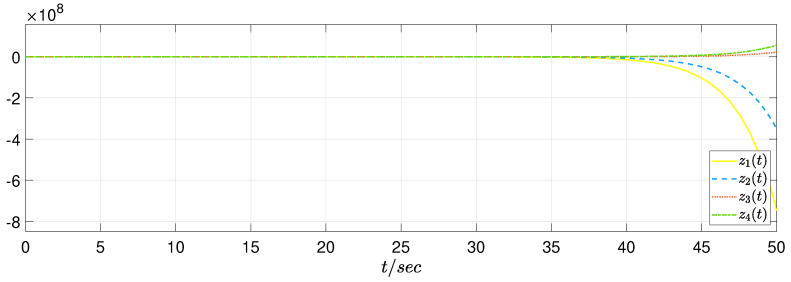
State response of original system without control.

**Figure 6:**
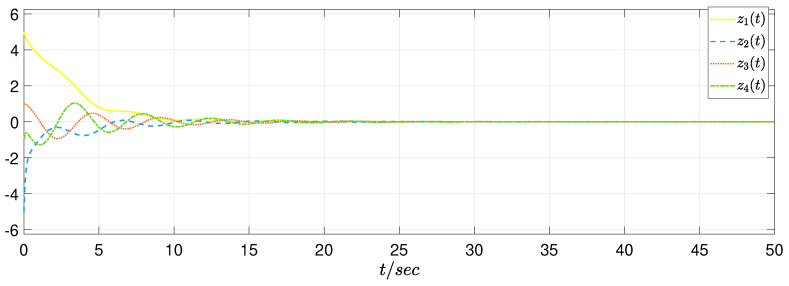
State response of closed-loop system.

**Figure 7:**
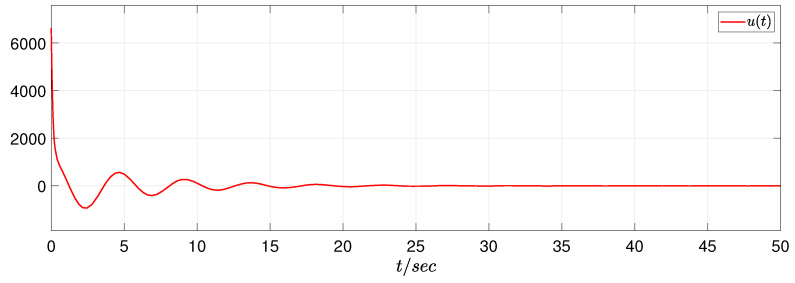
The SMC input.

In view of the simulation purpose, the initial condition is set as $\varphi \left(s\right)={\left[5\;\;-5\;\;1\;\;-1\right]}^{T}$ and $\psi \left(s\right)={\left[0\;\;0\;\;0\;\;0\right]}^{T}$, s∈[−0.2,0]. The unknown uncertainty and external disturbance are provided as $f\left(z\left(t\right)\right)=0.1\sin\left(z_{1}\left(t\right)\right)$ and $w\left(t\right)=[1/\left(t^{2}+1\right)\;\;1/\left(t^{2}+1\right)]$, respectively. In the RBF neural network, the width $\sigma_{j}=1$, the initial weight $\hat{\theta }\left(0\right)=0$, Λ=0.1, and the center point $m_{j}={\left[-1\;\;-0.5\;\;0\;\;0.5\;\;1\right]}^{T}$. In addition, the switching signal sgn(s(z,t)) is changed by s(z,t)/(∥s(z,t)∥+0.01) and ρ=0.01. Then, we have the simulation results as presented in Figure 5, Figure 6 and Figure 7. Figure 5 plots that the original system is unstable without control; Figure 6 depicts that the system achieves a stability property by the proposed control algorithm; Figure 7 gives the SMC input, which shows a satisfactory system performance is achieved.

**Figure 8:**
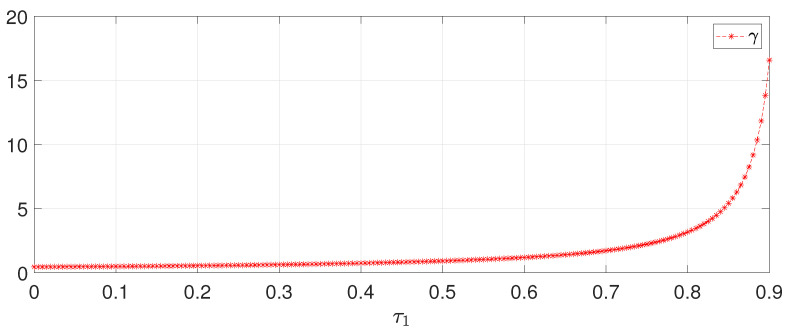
Minimum allowable γ for different time-delay.

Seen from Theorem 3, the index γ is sensitive to the system time-delay. To reflect the relationship between γ and $\tau_{1}$, the following Table 1 shows the maximum allowable values for $\tau_{1}$ when different values for γ are given in Theorem 3, in which it is seen that large γ allows bigger time-delay. Otherwise, to understand how the time-delay may affect γ, a trajectory is plotted in Figure 8 to show the minimum allowable γ for given $\tau_{1}$, and the result also reveals that large $\tau_{1}$ demands bigger γ.

## 5. Conclusions

The problem of SMC for passification of nonlinear time-delay systems via an adaptive neural network approach has been tackled in this paper. First, a mathematical model for the crane has been established based on the generalized Lagrange formula. Second, by proposing an integral-type sliding surface, on which the SMD with desirable dynamic property has been derived. Third, based on the RBF neural network approach, an adaptive SMC law has been synthesized to ensure the sliding motion in finite-time. Fourth, in order to check the passivity performance of the SMD system, feasible easy-checking LMI conditions have been developed. Finally, a numerical study with simulation has been put forward to verify the correctness of the proposed method.

## Figures and Tables

**Table 1:** Maximum allowable $\tau_{1}$ for different γ by Theorem 3.

γ	2	2.5	3	3.5	4	4.5	5
$\tau_{1}$	0.730	0.768	0.793	0.811	0.824	0.835	0.844

## Data Availability

Not applicable.

## References

[1] Jia X., Chen X., Xu S., Zhang B., Zhang Z. (2017). Adaptive output feedback control of nonlinear time-delay systems with application to chemical reactor systems. IEEE Trans. Ind. Electron..

[2] Yi S., Ulsoy A.G., Nelson P.W. (2010). Design of observer-based feedback control for time-delay systems with application to automotive powertrain control. J. Frankl. Inst..

[3] Boussaada I., Tliba S., Niculescu S.I., Ünal H.U., Vyhlídal T. (2018). Further remarks on the effect of multiple spectral values on the dynamics of time-delay systems. Application to the control of a mechanical system. Linear Algebra Its Appl..

[4] Gopalsamy K. (2013). Stability and Oscillations in Delay Differential Equations of Population Dynamics.

[5] Arik S. (2014). An analysis of stability of neutral-type neural systems with constant time delays. J. Frankl. Inst..

[6] Xu X., Liu L., Feng G. (2017). Consensus of heterogeneous linear multiagent systems with communication time-delays. IEEE Trans. Cybern..

[7] Wu M., He Y., She J.H., Liu G.P. (2004). Delay-dependent criteria for robust stability of time-varying delay systems. Automatica.

[8] Wu L., Su X., Shi P., Qiu J. (2010). A new approach to stability analysis and stabilization of discrete-time TS fuzzy time-varying delay systems. IEEE Trans. Syst. Man Cybern. Part B Cybern..

[9] Karimi H.R. (2011). Robust delay-dependent *H*_∞_ control of uncertain time-delay systems with mixed neutral, discrete, and distributed time-delays and Markovian switching parameters. IEEE Trans. Circuits Syst. I Regul. Pap..

[10] Feng Z., Lam J. (2012). Integral partitioning approach to robust stabilization for uncertain distributed time-delay systems. Int. J. Robust Nonlinear Control.

[11] Wang Y.E., Wu D., Karimi H.R. (2022). Robust stability of switched nonlinear systems with delay and sampling. Int. J. Robust Nonlinear Control.

[12] Xiao H., Zhu Q., Karimi H.R. (2022). Stability of stochastic delay switched neural networks with all unstable subsystems: A multiple discretized Lyapunov-Krasovskii functionals method. Inf. Sci..

[13] Wei Y., Karimi H.R., Yang S. (2022). New results on sampled-data output-feedback control of linear parameter-varying systems. Int. J. Robust Nonlinear Control.

[14] Utkin V.I., Vadim I. (2004). Sliding mode control. Var. Struct. Syst. Princ. Implement..

[15] Baek J., Jin M., Han S. (2016). A new adaptive sliding-mode control scheme for application to robot manipulators. IEEE Trans. Ind. Electron..

[16] Wang Y., Gao Y., Karimi H.R., Shen H., Fang Z. (2018). Sliding mode control of fuzzy singularly perturbed systems with application to electric circuit. IEEE Trans. Syst. Man Cybern. Syst..

[17] Wang Y., Jiang B., Wu Z.G., Xie S., Peng Y. (2020). Adaptive sliding mode fault-tolerant fuzzy tracking control with application to unmanned marine vehicles. IEEE Trans. Syst. Man Cybern. Syst..

[18] Wang Y., Feng Y., Zhang X., Liang J. (2019). A new reaching law for antidisturbance sliding-mode control of PMSM speed regulation system. IEEE Trans. Power Electron..

[19] Sumantri B., Uchiyama N., Sano S. (2016). Least square based sliding mode control for a quad-rotor helicopter and energy saving by chattering reduction. Mech. Syst. Signal Process..

[20] Utkin V. (2015). Discussion aspects of high-order sliding mode control. IEEE Trans. Autom. Control.

[21] Guo X., Liu Z., Gao C. (2022). Fault-tolerant adaptive control for uncertain switched systems with time-varying delay and actuator faults based on sliding mode technique. J. Frankl. Inst..

[22] Jiang B., Karimi H.R., Yang S., Gao C., Kao Y. (2020). Observer-based adaptive sliding mode control for nonlinear stochastic Markov jump systems via TšCS fuzzy modeling: Applications to robot arm model. IEEE Trans. Ind. Electron..

[23] Palraj J., Mathiyalagan K., Shi P. (2021). New results on robust sliding mode control for linear time-delay systems. IMA J. Math. Control Inf..

[24] Jiang B., Gao C.C. (2022). Decentralized adaptive sliding mode control of large-scale semi-Markovian jump interconnected systems with dead-zone input. IEEE Trans. Autom. Control.

[25] Liu Z., Karimi H.R., Yu J. (2020). Passivity-based robust sliding mode synthesis for uncertain delayed stochastic systems via state observer. Automatica.

[26] Jiang B., Wu Z., Karimi H.R. (2022). A distributed dynamic event-triggered mechanism to HMM-based observer design for *H*_∞_ sliding mode control of Markov jump systems. Automatica.

[27] Lewis F.W., Jagannathan S., Yesildirak A. (2020). Neural Network Control of Robot Manipulators and Non-Linear Systems.

[28] Vasickaninova A., Bakosova M., Meszaros A., Klemeš J.J. (2011). Neural network predictive control of a heat exchanger. Appl. Therm. Eng..

[29] Aguiar C., Leite D., Pereira D., Andonovski G., Skrjanc I. (2021). Nonlinear modeling and robust LMI fuzzy control of overhead crane systems. J. Frankl. Inst..

[30] Chen Y., Xue A., Lu R., Zhou S. (2008). On robustly exponential stability of uncertain neutral systems with time-varying delays and nonlinear perturbations. Nonlinear Anal. Theory, Methods Appl..

[31] Wu Z.G., Park J.H., Su H., Chu J. (2013). Delay-dependent passivity for singular Markov jump systems with time-delays. Commun. Nonlinear Sci. Numer. Simul..

[32] Gu K., Chen J., Kharitonov V.L. (2003). Stability of Time-Delay Systems.

